# Gut microbiome diversity of porcine peritonitis model of sepsis

**DOI:** 10.1038/s41598-022-21079-6

**Published:** 2022-10-19

**Authors:** Miroslava Chalupova, Jan Horak, Lenka Kramna, Lukas Nalos, Milan Stengl, Katerina Chudejova, Lucie Kraftova, Ondrej Cinek, Pavel Klein, Martin Matejovic, Jaroslav Hrabak

**Affiliations:** 1 Biomedical Center, Faculty Medicine in Pilsen, Charles University, alej. Svobody 76, 323 00 Pilsen, Czech Republic; 2 Department of Stomatology, Faculty Medicine in Pilsen, Charles University, Pilsen, Czech Republic; 3 Department of Internal Medicine, Faculty Medicine in Pilsen, Charles University, Pilsen, Czech Republic; 4 Department of Paediatrics, 2nd Faculty of Medicine, Charles University and University Hospital Motol, Prague, Czech Republic; 5 Department of Physiology, Faculty Medicine in Pilsen, Charles University, Pilsen, Czech Republic; 6 Department of Microbiology, Faculty Medicine in Pilsen, Charles University, Pilsen, Czech Republic

**Keywords:** Microbiome, Outcomes research, Preclinical research, Translational research

## Abstract

Animal models are essential in understanding of the mechanisms of sepsis moreover the development and the assessment of emerging therapies. In clinically relevant porcine model, however, a significant variability in the host response has been observed among animals. Thus, there is a strong demand to better understand the potential sources of this heterogeneity. In this study, we compared faecal microbiome composition of 12 animals. Three samples were collected at different time points from each animal. Bacteriome was subjected to 16S rDNA profiling. A significant difference in bacterial composition was associated with the season (p < 0.001) but not with the sex of the pig (p = 0.28), the timing of sample collection (p = 0.59), or interactions thereof (all p > 0.3). The season batch explained 55% of the total variance in the bacteriome diversity. The season term was highly significant from the high-resolution level of the bacterial amplicon sequencing variants up to the level of phylum. The diversity of the microbiome composition could significantly influence experimental model of sepsis, and studies are warranted to demonstrate the effects of gut microbiome diversity on the host-response. If confirmed, control of the gut microbiome should become a standard part of the pre-clinical sepsis experiments.

## Introduction

Sepsis, a syndrome defined as life-threatening organ dysfunction caused by dysregulated host response to infection^1^, represents a serious threat for current medicine with predicted annual mortality of 5.3 million patients worldwide^2^. Thus, the basic research, the therapeutical interventions and the epidemiological studies on sepsis are of utmost clinical importance^3–6^. Several animal models have been developed to study the pathogenesis of sepsis. Among them, mice and rodent models seems to be the least appropriate choice due to their immunological differences to humans, representing a limited translation potential. The more appropriate models, such as rabbits, pigs, monkeys and baboons provide some advantages, e.g., similar sensitivity and response to bacterial infection as in humans.

The porcine peritonitis model resulting in a sepsis show significant morbidity and mortality rates comparable with the human disease^7,8^. Peritonitis can be induced by in vitro grown bacteria (single species or polymicrobial) or by faecal soilage, usually by an autologous faecal inoculum^7,9^. Comparing both approaches, the pure culture-induced model demonstrates initial acute peritonitis and damaging effects correlating with higher level of circulating lipopolysaccharide. The faecal soilage-induced model shows acute peritonitis phase with pronounced peritoneal reaction with the signs of developed systemic inflammatory response^9^. Even though the latter model shows high translation potential, significant variability has been described among various studies^7^. We hypothesize that the variability can be due to differences in the immune response among the animals and/or due to differences in their gut microbiome.

As the pigs represent one of the most important animal models, several studies have focused on their microbiome^10^. Recently, the dynamics of porcine gut microbiome in the acute phase of ischemic stroke has been described^11^. Beta-diversity of the microbiome showed the changes in the acute phase of the stroke, but the microbiome pattern returned to the pre-experimental values, demonstrating the high stability of the porcine microbiome.

However, to our knowledge, there is no similar study on the gut microbiome of porcine model of sepsis. Thus, our study focused on the dynamics of the gut microbiome diversity in the pigs during their stay in the animal experimental facility, from their admission until the time of the experiments.

## Materials and methods

### Experimental model

Animal handling was performed according to the European Directive for the Protection of Vertebrate Animals Used for Experimental and Other Scientific Purposes (86/609/EU). Samples collection from the animals followed rules approved by the Animal Welfare Advisory Committee at the Ministry of Education, Youth and Sports of the Czech Republic (approval ID: MSMT-20064/2015-3 and MSMT-7311/2018-4) and was conducted under supervision of the Animal Welfare Advisory Committee at the Charles University, Faculty of Medicine in Pilsen.

Twelve domestic pigs of either sex were used. The animals were obtained from the animal farm specialized in breeding of Czech national Black Spotted Presticke breed of the average weight of 44.5 kg between January and November of 2018. The animals received care according to EU directive 63/2010. Both, on the home farm and in experimental conditions, the animals had a light cycle of 12 h/12 h, free access to water and were fed twice a day with feed from the same producer (complete feed mixtures A1 up to 28 kg and CDP from 29 kg; Zeten Blovice, Czech Republic).

### Samples

From each animal, three specimens of stool were collected (Table 1). The first sample was collected immediately from the first defecation of the animal in the housing pens after the admission from the animal farm. The second sample was collected before the experiment started after housing of the animal in the animal facility. Before the induction of peritonitis, approximately 100 g of the autologous stool sampe collected from the animal in 100 ml of isotonic saline was incubated at 37 °C during 10-h recovery period as previously described elsewhere^12^. To determine possible change of microbiome composition during the incubation period, the third sample was collected from the culture. Approximately 30 g of the stool were collected and stored in sterile plastic container at − 80 °C prior to DNA isolation.

**Table 1:** Admission of experimental animals and the time of their housing in animal facility.

Pig No.	Date of admission to the facility	Date of the experiment	Days spent in housing pen	Sex
1	19. 1. 2018	22. 1. 2018	3	F
2	8. 2. 2018	12. 2. 2018	4	F
3	8. 2. 2018	19. 2. 2018	11	F
4	8. 2. 2018	26. 2. 2018	18	M
5	16. 5. 2018	28. 5. 2018	12	M
6	16. 5. 2018	29. 5. 2018	6	F
7	29. 5. 2018	4. 6. 2018	7	F
8	29. 5. 2018	5. 6. 2018	13	F
9	3. 10. 2018	23. 10. 2018	20	M
10	26. 10. 2018	30. 10. 2018	4	M
11	26. 10. 2018	6. 11. 2018	11	M
12	26. 10. 2018	13. 11. 2018	18	F

### Extraction of DNA, PCR amplification and amplicon sequencing

Bacteriome profiling was performed according to Kozich et al.^13^ Ten grams of stool samples were homogenized by mixing and the DNA was extracted by NucleoSpin Soil isolation kit (Macherey–Nagel, Dueren, Germany). To avoid intralaboratory variability, DNA isolation was perfomed simultaneously by the same technician, same DNA isolation kit at the same time. Positive and negative controls were included during the whole analysis, including DNA extraction process. The variable region 4 of the 16S bacterial ribosomal gene was amplified by AccuPrime *Pfx* SuperMix (Invitrogen, Waltham, MA) with initial denaturation at 95 °C for 5 min, followed by 30 cycles of denaturation at 95 °C for 15 s, primer annealing at 55 °C for 30 s and extension at 68 °C for 1 min. The products were visualized on a 1.5% agarose gel and purified by AMPure XP purification kit (Beckman Coulter, Fullerton, CA). After equalizing and pooling the samples, the library was supplemented with 20% PhiX spike-in for balancing the signal, and sequenced on a MiSeq instrument using a MiSeq Reagent Kit v2 (500 cycles) sequencing kit (both Illumina Inc., San Diego, CA).

As some significant pathogens (*Chlamydia suis*, and the family *Erysipelotrichaceae*) have been identified in 16S rRNA profiling in operational taxonomic units (OTUs), specific PCR for detection of *Ch. suis*, *E. rhusiopathiae* and *E. tonsillarum* was performed^14,15^.

### Bioinformatic and statistical analysis

Sequencing data were trimmed, filtered and processed using DADA2 pipeline (version 1.16)^16,17^ in RStudio (version 1.3.1073)., with read length trimmed according to sequence quality, and chimeras removed.

Sequences were taxonomically classified using SILVA (version 128) database^18^. Amplicon sequence variants (analogous to operational taxonomic units, OTUs) were then inspected in MicrobiomeAnalyst^19^, and further analysed using vegan^20^ and phyloseq^21^ in R.

The read sets were rarefied to 100,000 per sample, and alpha diversity characterized using observed species counts, Chao1, Shannon and Simpson indices; their relation to potential predictors was assessed using linear models. Rare OTUs were then filtered, keeping only those being present at more than 0.1% in more than three samples. The relative composition of bacteriomes was plotted as compositional plots. The predictors of the composition were assessed using constrained ordination, the Hellinger transformation-based redundancy analysis, and their significance tested using permutation methods (commands rda and anova in *vegan*).

Beta diversity (dissimilarity between communities) was assessed using the quantitative Bray–Curtis index. Ordination was performed using non-metric multidimensional scaling (NMDS). After visual inspection of ordination graphs, the separation of the bacteriome profiles by predictors (season, type of sample, sex of the animal and interactions) was tested by Permutational Multivariate Analysis of Variance (PERMANOVA) implemented in the *vegan* package as the *adonis* function, with 1000 permutations. Lack of difference in variances among groups was verified by the function *betadisper*.

### Ethical approval

Institutional Review Board Statement: Animal handling was performed according to the European Directive for the Protection of Vertebrate Animals Used for Experimental and Other Scientific Purposes (86/609/EU). The experimental protocol was approved by the Committee for Experiments on Animals of the Faculty of Medicine in Pilsen, Charles University. The facility is approved by Ministry of Agriculture of the Czech Republic for performing animal experiments (Nr. 3456/2021-MZE-18134).

## Results

A total of 34 samples were analysed; two samples (pig Nr. 1 after admission and pig Nr. 4 before experiment) were lost during processing. The total number of reads ranged between 180 000 and 426 000. Prior to analyses, samples were rarefied to 100,000.

The alpha (intra-sample) diversity upon admission differed by season in all assessed indices (p < 0.05 for all, Supplementary Fig. 1), whereas sex was not associated. There was no systematic change in alpha diversity over the course of the experiment (admission—experiment—incubation bacteriomes, paired tests).

**Figure 1:**
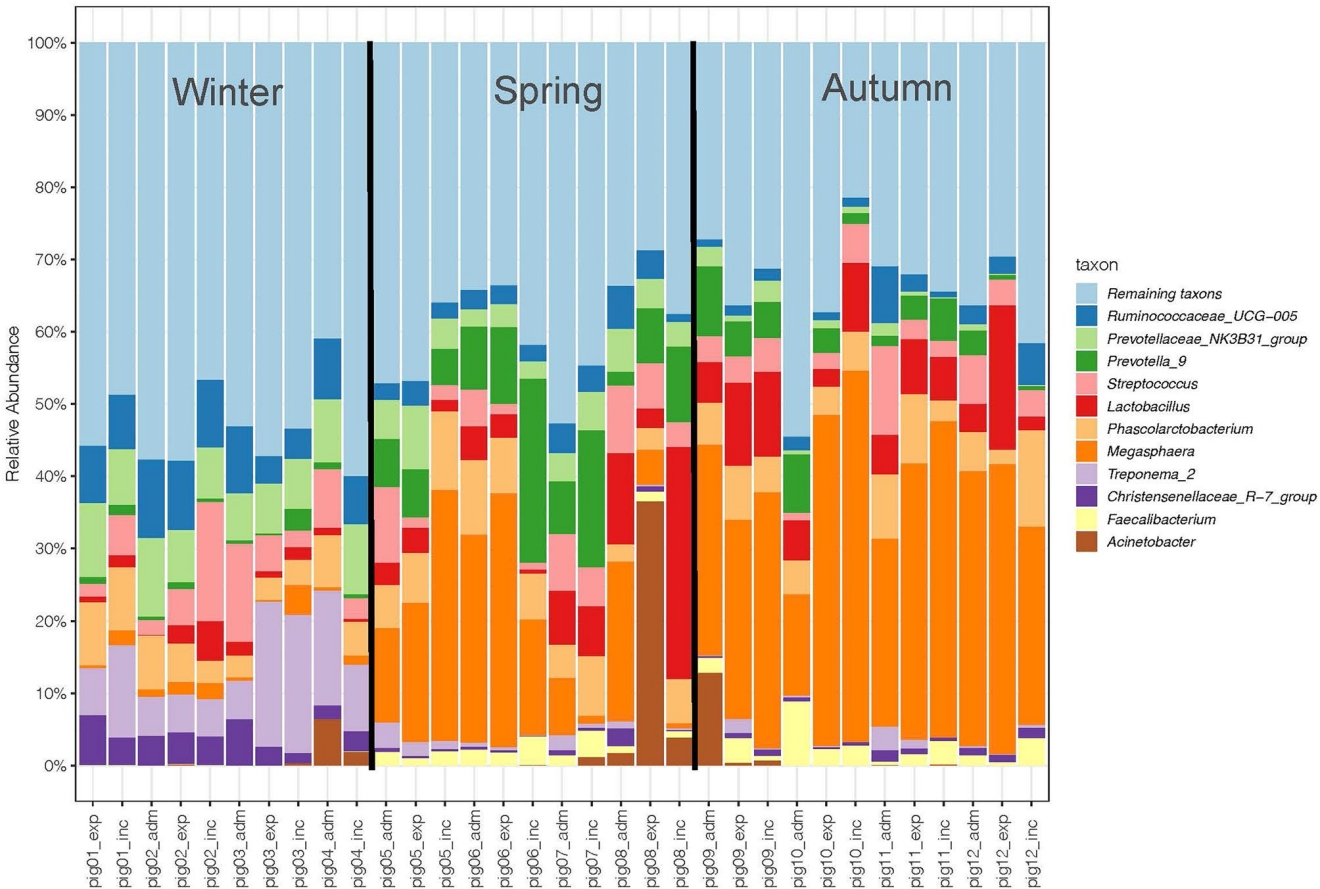
Taxonomic composition of bacteriomes showing predominant taxa at the genus level plotted as a compositional bar plot. For detailed genus composition, see Supplementary Fig. 2E. Microbiomes of the pigs are plotted at the time of admission (“pig*Nr*_adm”), immediately before the experiment (“pig*Nr*_exp”), and after incubation in isotonic saline before peritonitis induction (“pig*Nr*_inc”)—x-axis. Time between the admission of experimental animal into the animal facility and the experiment is listed in Table 1.

Abundance of individual taxa in the bacteriomes is plotted as a compositional bar plot (Fig. 1 with leading genera) and shown in heatmap tables (Supplementary Fig. 2A–E with prominent taxa at the level of phylum, class, order, family, and genus). At the order level, the most abundant taxa were Clostridiales, Selenomonadales, Bacteroidales and Lactobacillales. At the family level, the most frequent were *Veillonellaceae*, *Ruminococcaceae*, and *Prevotellaceae*.

Compositional plots are shown at the genus level, depicting predominant genera. Heterogeneity of composition among seasons is apparent already upon visual inspection.

**Figure 2:**
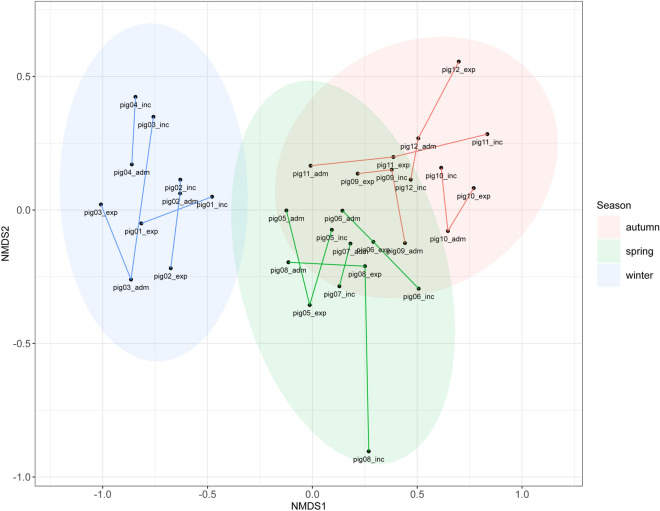
Non-metric multidimensional scaling on the quantitative Bray–Curtis dissimilarity (beta diversity—dissimilarity between communities). Testing was performed by PERMANOVA at the genus level. Pigs are identified according to their numbers and the time of their admission into the animal facility (“pig*Nr*_adm”), immediately before the experiment (“pig*Nr*_exp”), and after incubation in isotonic saline before peritonitis induction (“pig*Nr*_inc”). Detailed information describing the time of animal housing in the pen is listed in Table 1.

We then calculated the beta diversity (between samples) of the bacteriome using the Bray–Curtis dissimilarity on double-Wisconsin transformed data agglomerated at the taxonomic level of genus. Its ordination was performed using NMDS (Fig. 2, non-metric fit between observed dissimilarity and ordination distance had R^2^ = 0.991 with stress of 0.096). There were three visually identifiable clusters corresponding to the seasonal batches of experimental pigs.

The formal testing of differences among group means of beta diversity was done by PERMANOVA at the level of genus. It showed that samples clustered significantly by season (p < 0.001) but not with sex of the pig (p = 0.28), the type of sample (p = 0.59), or interactions thereof (all p > 0.3). The season batch explained 55% of the total variance in bacteriome diversity. When constructing the model at other taxonomic levels than genus, the season term was highly significant from the high-resolution level of bacterial amplicon sequencing variants up to the level of phylum. The respective terms in the PERMDISP tests of homogeneity of multivariate variances were all statistically insignificant, the variances did not differ in any of the compared predictors (data not shown), which indicated that the above association observed in PERMANOVA was due to a genuine difference between the position of *season* centroids, rather than due to differences in spread.

The predictors of bacteriome community composition were then tested using constrained ordination, the redundancy analysis on the Hellinger-transformed abundance data. We arrived at analogous results: season was a significant predictor (p < 0.001), but not sex of pig or type of samples.

After 10 h incubation of the stool in isotonic saline prior peritonitis induction, the bacterial composition remains very similar showing no significant enrichment of specific taxa. The viability of the bacteria, however, could not be determined as most taxa are fastidious or unculturable.

In 26 samples, sequences corresponding to significant porcine pathogen, *Chlamydia suis,* have been suggested among the rDNA sequencing signal. As this bacterium could significantly influence the severity of the sepsis, we decided to confirm or refute the presence of this bacterium by specific PCR. Similarly, other potential pathogens from the *Erysipelotrichaceae* family were tested. Specific PCR, however, identified none of those pathogens.

## Discussion

As demonstrated by several studies, the sepsis modelling using porcine peritonitis induced by autologous faecal soilage shows significant variability^7^. To eliminate such a variability, standardization of the experiments requires at least the use of genetically closely related animals and characterization of inoculum used for sepsis induction.

As demonstrated in our data, microbiome composition significantly differs in the pigs from the same breeding facility between the winter period comparing with the spring and autumn period. No significant change of microbiomes was observed during the animal housing in our facility (see Figs. 1 and 2). That observation may be due to relatively short time of animals’ housing in the pen (see Table 1) or due to restricted contact among the animals. Composition of present microbiomes mainly differed with marked presence of *Treponema* spp. during winter period and *Megasphaera* spp. in spring and autumn period. All the pigs were obtained from the same farm with closely related population, receiving the same feed in the farm as well as in the animal facility during the study period. Similarly, the welfare conditions were the same in all the periods. We also received negative information from the farm as to antibiotic therapy or prophylaxis. Therefore, it is difficult to find any specific parameter that could be responsible for microbiome differences in the animal farm.

Looft et al.^22^ performed a deep study on microbiome of different parts of gastrointestinal tract of pigs with and without antibiotics using sequencing of V1-V3 16S rRNA region and by metagenomics. They studied microbial communities from small (ileum) and large intestine (cecum and colon) and the faeces with and without antibiotic pressure. Interestingly, they described a large difference in microbiome compositions in different anatomical location. In the mid colon and faeces, they found abundance of Firmicutes and Bacteroidetes taxa following by Proteobacteria and Spirochaetes which is in accordance with our results (see Supplementary Fig. 2A). They also found the significant presence of *Treponema* spp. in the pigs that were not treated with antibiotics.

Recently, Xiang et al.^23^ published a microbiome analysis of five species used for experimental research including mini pigs. They found the microbiome composition similar to our study with the predominance of Firmicutes and Bacteroidetes and significantly lower percentage of Actinobacteria compared to the human microbiota.

In the porcine peritonitis model, systemic inflammatory response is induced using the faecal soilage containing several microbial species^24–26^. Only few bacteria, however, have been identified in blood using a cultivation method, mainly *Escherichia coli*, *Streptococcus* spp., *Enterococcus* spp. and no commonly present faecal bacteria, e.g., *Clostridium* spp. or *Bacteroides* spp. that are present in high abundance in the faecal soilage used for peritonitis induction. The lack of those bacteria can be due to their lower ability for the invasion and surviving in oxygen-rich blood or due to overgrowing of Enterobacterales in blood cultures due to their simple nutritional requirements. The findings are similar with human peritonitis with predominance of *Escherichia* coli, other Enterobacterales, *Staphylococcus* spp. and *Streptococcus* spp.^27^.

The 16S rRNA profiling does not allow to describe bacterial communities at the species or even subspecies level, including bacterial toxin producers. Therefore, some important pathogens can be missed by that approach. By the deep analysis of OTUs, we identified putative sequences of some significant porcine pathogens (e.g., *Chlamydia suis*, *Erysipelothrix* spp.) in majority of the samples. Using specific PCR, however, we detected none of those pathogens. Finding such sequences in raw data can be due to 16S rDNA marker sequences shared with other, harmless species that profiling of short fragments cannot distinguish.

Taken together, based on beta-diversity analysis of the samples, we described distinct clusters in bacterial populations in experimental pigs during winter and spring/autumn period. No significant change was observed during the holding period in the pens or after incubation of bacteria in the isotonic saline before their inoculation into peritoneum. Further studies should focus on the clinical relevance of microbiome composition in the peritonitis model using faecal soilage as our data demonstrate significant differences in bacterial communities. Nevertheless, determining the role of gut microbiota diversity in animal models could become an essential component in pre-clinical sepsis research to sustain their reproducibility, especially in models using autologous inoculation of microbiota.

## Supplementary Information

Supplementary Legends.

Supplementary Figure 1.

Supplementary Figure 2.

Supplementary Figure 2.

Supplementary Figure 2.

Supplementary Figure 2.

Supplementary Figure 2.

## Data Availability

All data are available as supplementary material. Raw sequencing data has been deposited to the Sequence Read Archive (SRA) under BioProject PRJNA807586.
