# Supplementary material for: Gut microbiome diversity of porcine peritonitis model of sepsis
Source: Sci Rep. 2022 Oct 19;12:17430. doi: 10.1038/s41598-022-21079-6 (PMC9581925; doi:10.1038/s41598-022-21079-6)
Supplement: Supplementary file 1 — Supplementary Legends. [file 41598_2022_21079_MOESM1_ESM.docx]

**Supplementary Figure 1.** Alpha diversity of the bacteriomes by season.

**Supplementary Figure 2.** Most abundant taxa and their proportions in studied samples. Taxa are selected by average proportions in the whole study: A) at the phylum level, B) at the class level, C) at the order level, D) at the family level, E) at the genus level.

The figures were created by a custom script written in the R programming language (R version 4.1.2, https://www.R-project.org/), kindly see the *Bioinformatic and statistical analysis* section of the main document for details.
