# Supplementary figures and images for: Gut microbiome diversity of porcine peritonitis model of sepsis
Source: Sci Rep. 2022 Oct 19;12:17430. doi: 10.1038/s41598-022-21079-6 (PMC9581925; doi:10.1038/s41598-022-21079-6)

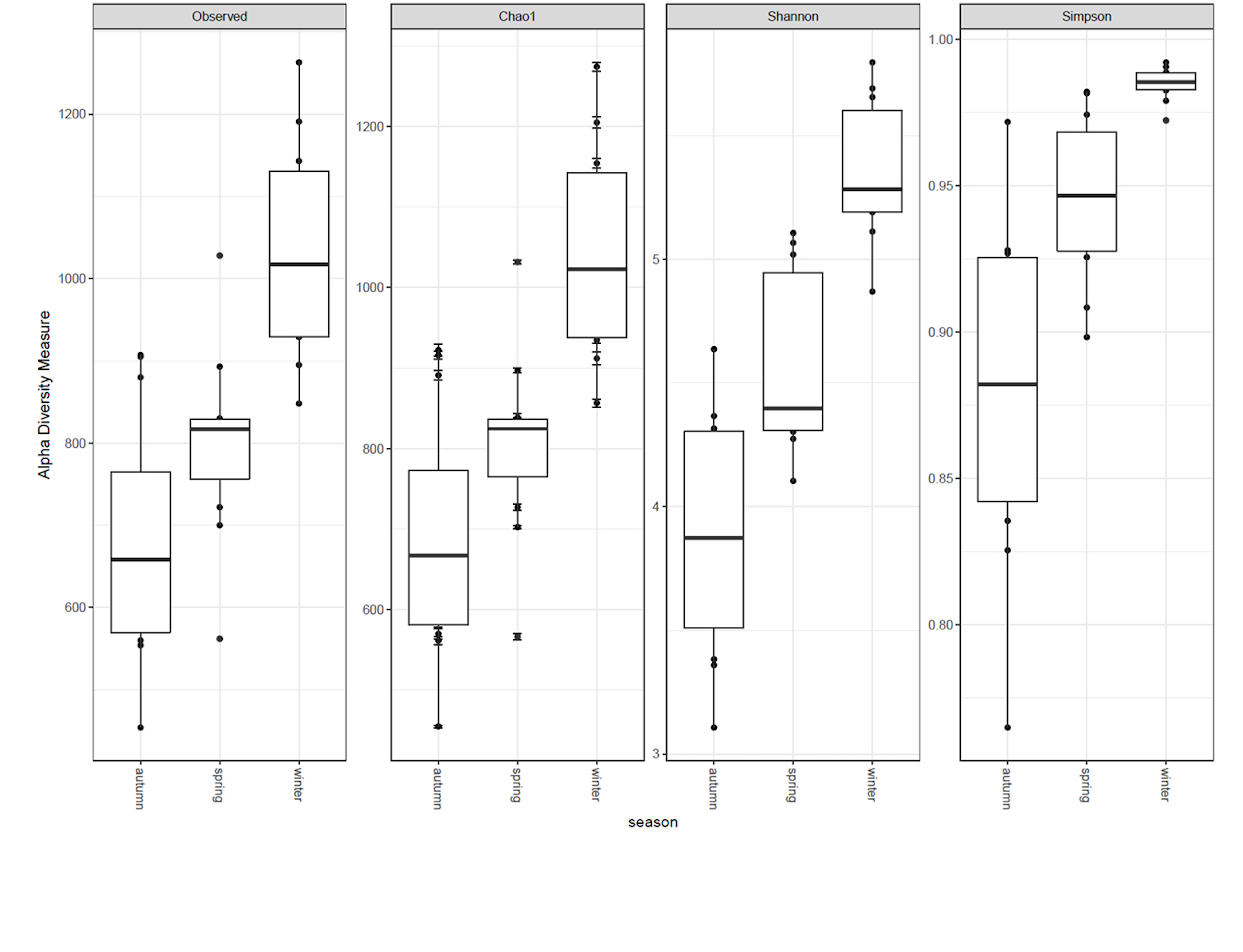

Supplement: Supplementary file 2 — Supplementary Figure 1. [file 41598_2022_21079_MOESM2_ESM.jpg]

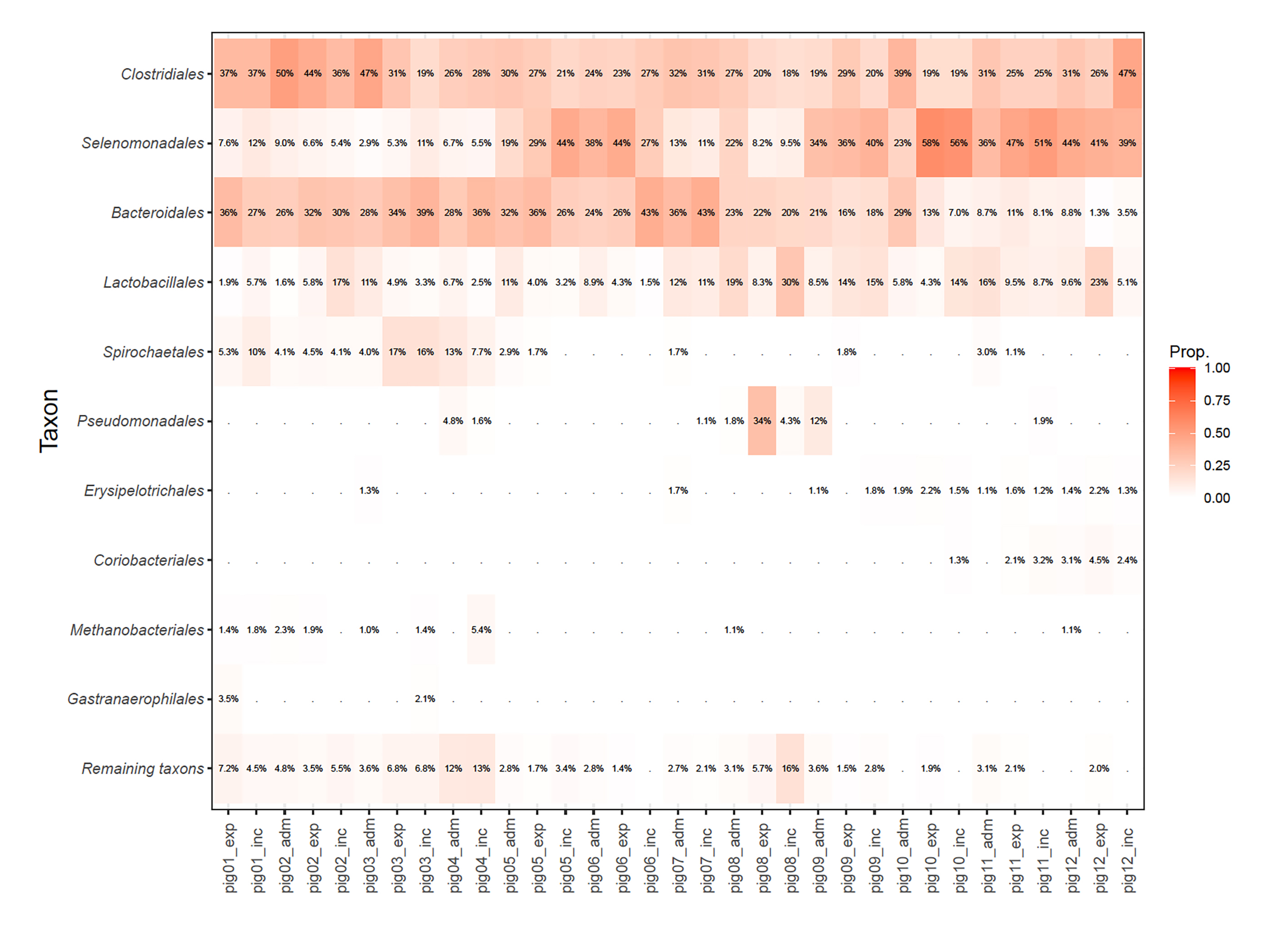

Supplement: Supplementary file 5 — Supplementary Figure 2. [file 41598_2022_21079_MOESM5_ESM.jpg]

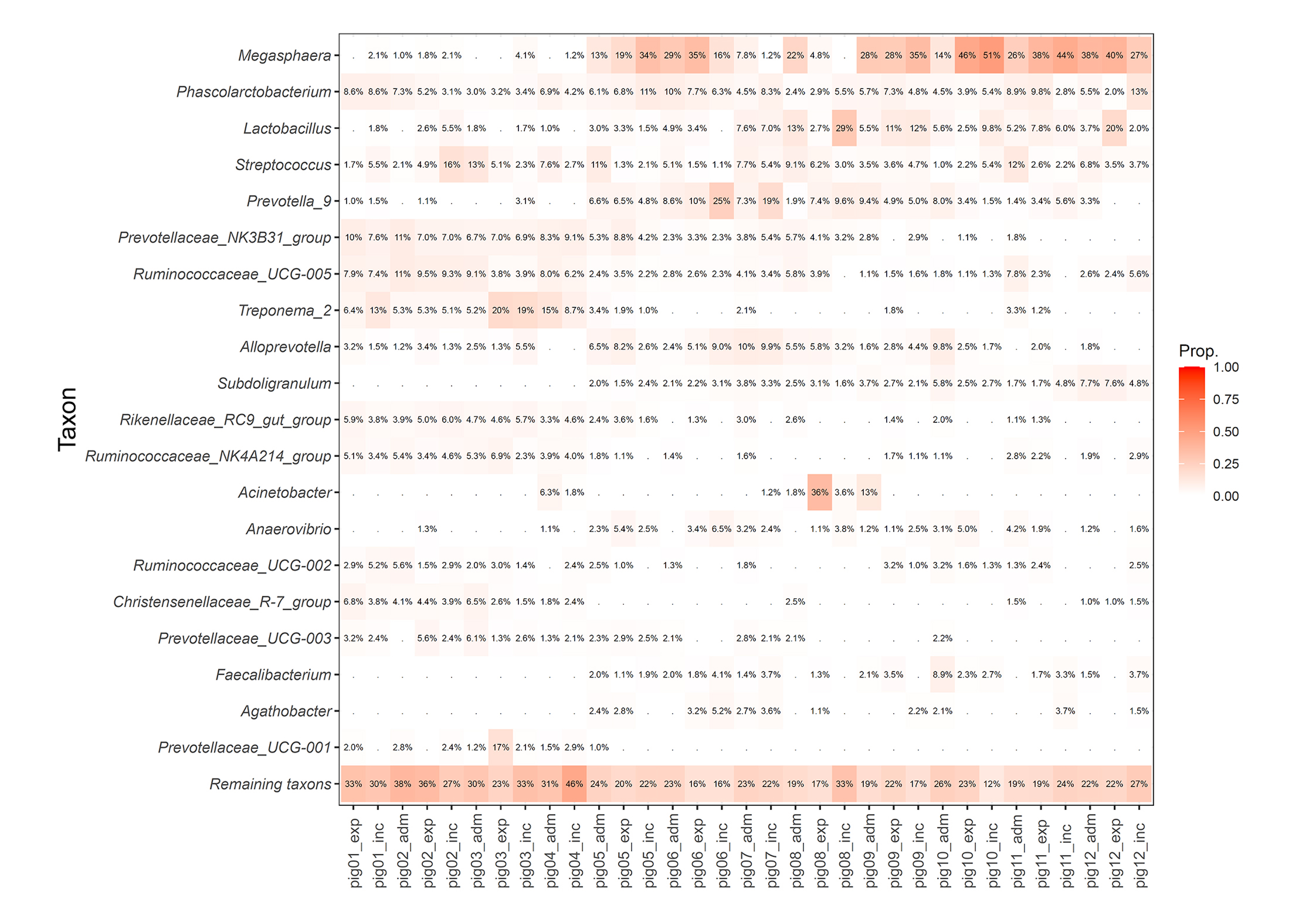

Supplement: Supplementary file 7 — Supplementary Figure 2. [file 41598_2022_21079_MOESM7_ESM.jpg]
